# TIME COURSE FOR ACQUIRING TOILETING INDEPENDENCE IN PATIENTS WITH SUBACUTE STROKE: A PROSPECTIVE COHORT STUDY

**DOI:** 10.2340/jrm.v57.42390

**Published:** 2025-05-20

**Authors:** Shin KITAMURA, Yohei OTAKA, Shintaro UEHARA, Yudai MURAYAMA, Kazuki USHIZAWA, Yuya NARITA, Naho NAKATSUKASA, Daisuke MATSUURA, Rieko OSU, Kunitsugu KONDO, Sachiko SAKATA

**Affiliations:** 1 Department of Rehabilitation Medicine, Tokyo Bay Rehabilitation Hospital, Chiba; 2 Faculty of Rehabilitation, School of Health Sciences, Fujita Health University, Aichi; 3 Department of Rehabilitation Medicine, School of Medicine, Fujita Health University, Aichi; 4 Faculty of Rehabilitation, School of Health Sciences, Chiba Prefectural University of Health Sciences, Chiba; 5 Faculty of Human Sciences, Waseda University, Saitama, Japan

**Keywords:** activities of daily living, cerebrovascular disorders, cluster analysis, task performance, wheelchair

## Abstract

**Objective:**

To determine the time course of longitudinal changes in the independence level of toileting-related subtasks in post-stroke patients.

**Design:**

Single-institution, prospective cohort study.

**Subjects/Patients:**

A total of 101 consecutive patients with stroke admitted to subacute rehabilitation wards who urinated/defecated in bathrooms using wheelchairs upon admission.

**Methods:**

Occupational therapists assessed the independence level of patients in each of the 24 toileting subtasks on a 3-level rating scale using the Toileting Tasks Assessment Form every 2–4 weeks from admission to the endpoint (achieving independent toileting or discharge). Patients were classified based on admission and endpoint assessment form scores using a two-step cluster analysis.

**Results:**

Patients were classified into Cluster 1 (30 patients who exhibited a greater independence level in all subtasks upon admission [46.7–100% of patients performed each subtask independently] to the endpoint [73.3–100%]), Cluster 2 (41 patients who showed less independence upon admission [0–26.8%] but gained greater independence at the endpoint [34.1–73.2%]), and Cluster 3 (30 patients whose independence levels remained low in many subtasks from admission [0–26.7%] to the endpoint [3.3–26.7%]).

**Conclusion:**

Changes in toileting independence levels could be classified into 3 time courses. Effective intervention strategies may differ between each group.

## LAY ABSTRACT

For patients after a stroke, acquiring toileting independence is critical in rebuilding their lives. Toileting consists of multiple subtasks, including entering and exiting bathrooms, transferring from the wheelchair to the toilet seat, and manipulating lower garments. Understanding the potential processes towards independence in toileting subtasks helps therapists prioritize subtasks for patients to practise and develop effective strategies to promote independence. We examined the changes in the independence level of toileting-related subtasks in 101 stroke patients and found that the process can be classified into 3 time courses as follows: patients who were nearly independent in many subtasks upon admission and became independent early during hospitalization; patients who required assistance with many subtasks upon admission but could perform many subtasks independently during their hospitalization; and patients who required assistance upon admission and remained dependent on discharge. Different intervention strategies are required for each group to facilitate the acquisition of toilet-related tasks.

For patients hospitalized after a stroke, acquiring the ability to perform toileting is critical to rebuilding their lives. Assisting with toileting is a psychological burden for both the patient after a stroke (1) and their caregivers (2). Toileting independence is a determinant of hospital discharge to home (3, 4) and a primary goal of rehabilitation (5). As gaining independent walking is challenging for patients with stroke (6–9), patients start practising toileting while still using wheelchairs. Therefore, strategies for wheelchair-using patients with stroke to acquire toileting independence are particularly important.

The effectiveness of various types of interventions has been demonstrated for overall activities of daily living (ADL) in patients with stroke (10–14); however, no effective strategy has been established for acquiring toileting for wheelchair users. When considering strategies to facilitate the acquisition of skills for toileting tasks, it is necessary to understand the independent processes of the subtasks that comprise toileting. Toileting is a serial task comprising multiple subtasks (15), such as entering and exiting the bathroom, transferring from the wheelchair to the toilet seat, and manipulating lower garments (16, 17). In our previous study, we identified 24 toileting subtasks and developed the Toileting Tasks Assessment Form (TTAF): a tool to assess the independence level for each subtask (16). Each subtask is an independent activity (16) with varying degrees of difficulty (18). Therefore, overall toileting independence is limited by which necessary subtasks have not been acquired, and the process towards acquiring full independence depends on the time course of acquiring independence in each subtask.

Patients with stroke exhibit diverse clinical characteristics influenced by the location or volume of brain damage (19–21), and demographic characteristics, such as age and sex. Therefore, the processes towards independence may also show different time courses owing to the interaction between the difficulty of each element of toileting skill (18) and individual clinical and demographic characteristics. Our previous study found 3 different time courses of bed–wheelchair transfer in patients after stroke and different characteristics of the patient groups exhibiting each time course (22). The time courses and their characteristics may be different between transferring and toileting because the difficulty of acquiring independence is different between these 2 tasks (6–9). If the independence process also differs in toileting, tailored rehabilitation strategies are essential for patients with different time courses of performance improvement. Patients who anticipate difficulty acquiring the required independence level may prioritize simpler subtasks and utilize compensatory measures, e.g., assistive devices, for difficult subtasks. Understanding the potential processes towards independence in toileting subtasks and identifying specific subtasks that tend to remain dependent can help therapists prioritize subtasks for patients to practise.

This prospective cohort study aimed to clarify the differences in the time course and factors associated with independence in toilet subtasks in patients after stroke. We classified patients into subtypes based on the time course of changes in the independence level of subtasks during hospitalization and investigated the characteristics of their time course and demographics within each subtype.

## METHODS

### Study design and setting

This single-centre prospective cohort study conformed to the Strengthening the Reporting of Observational Studies in Epidemiology Statement guidelines (23). The study was conducted in the Kaifukuki Rehabilitation Ward (KRW) of Tokyo Bay Rehabilitation Hospital, a Japanese 160-bed rehabilitation hospital. The KRW provides subacute intensive rehabilitation covered by Japan’s medical insurance system. Patients with stroke are eligible for admission within 2 months of stroke symptom onset and can stay for up to 6 months after admission (24). At this hospital, all patients received 2–3 h of daily rehabilitation sessions. As this is an exploratory study, the sample size was determined based on the planned study period (June 2016 to May 2017). The Ethics Committee of Tokyo Bay Rehabilitation Hospital approved the study protocol (approval no. 140), and all patients provided written informed consent before they participated in the study.

### Participants

Patients admitted to Tokyo Bay Rehabilitation Hospital after strokes were consecutively recruited between June 2016 and May 2017. The inclusion criteria comprised first ischaemic or haemorrhagic stroke, hemiparesis with apparent unilateral motor paresis on the motor items in the Stroke Impairment Assessment Set (SIAS) upon admission to the KRW, use of wheelchair for mobility upon admission, and provision of consent by themselves or via their legal representative. The exclusion criteria comprised the inability to urinate or defecate in the bathroom and toileting independence on admission.

### Procedure

An occupational therapist in charge of each participant assessed the independence level of toileting by observing the actual performance using the TTAF (16). The TTAF is a tool specifically developed to assess the toileting ability of patients who have experienced a paretic stroke (Fig. S1). The TTAF classifies a series of toileting tasks into 24 subtasks (Table I). The independence level of each subtask is assessed on a 3-point scale: 3 = independent (participant can complete the task by themselves without any assistance from a therapist); 2 = requiring supervision or verbal assistance (participant can complete a task under supervision or with verbal assistance by a therapist); 1 = requiring physical assistance (participant needs physical assistance from a therapist, such as locking the wheelchair brakes and manoeuvring the wheelchair to the appropriate position) to complete the task; and N = not applicable (participant does not need to perform the task: for example, the task of “put the foot on the footrest” that can be applied only to those using wheelchairs with footrests). The mean score of the subtasks was calculated by dividing the total score by the number of subtasks (excluding subtasks marked as not applicable [N]). This assessment demonstrated good reliability and validity (16).

**Table I:** Subtasks comprising the Toileting Tasks Assessment Form (TTAF)

Toileting task	Subtask
Wheelchair to the toilet seat	Open and close the door
	Manoeuvre the wheelchair toward the appropriate place for transfer to the toilet seat
	Lock the wheelchair brakes
	Press the nurse call button (before urination/defecation)
	Take the foot off the footrest and place it on the ground
	Stand up from the wheelchair
	Turn while standing (before urination/defecation)
	Maintain a standing position (before urination/defecation)
	Pull the lower garments down
Performance on the toilet seat	Sit on the toilet seat
	Maintain a sitting position on the toilet seat
	Dispose of incontinence pad/sanitary items
	Clean up after urination and/or defecation
	Flush the toilet
	Press the nurse call button (after urination/defecation)
Toilet seat to the wheelchair	Stand up from the toilet seat
	Maintain a standing position (after urination/defecation)
	Pull the lower garments up and adjust them
	Turn while standing (after urination/defecation)
	Sit on the wheelchair seat
	Put the foot on the footrest
	Unlock the wheelchair brakes
	Open and close the door
	Exit the toilet room

The items are listed in the order of their performance.

The assessments using the TTAF were performed by 39 occupational therapists affiliated with the hospital. The occupational therapists used the tool in their daily clinical practice and had been instructed on the purpose of the TTAF and how to score it. During assessment, participants were instructed to perform a series of toileting steps, from entering the bathroom to urinating or defecating, and then exiting the bathroom, using the same environmental settings and procedures as usual. In this hospital, patients typically used wheelchairs with flip-up arm supports and removable foot supports. In all the bathrooms, vertical and horizontal handrails were installed on the wall to the side of the toilet seat. Participants were assessed and practised in the environment selected by their therapists to ensure the highest level of participant independence.

For each assessment time point, the observational assessment was repeated on 3 different days. The lowest score of the 3 was used as the one representing the independence level of the subtask (if a participant’s performance for one subtask was scored as “3”, “3”, and “2”, then “2” was adopted). Assessments were conducted upon admission to KRW, 2 and 4 weeks after admission, and at 4-week intervals thereafter until reaching one of the following endpoints: (*i*) Independence, when all subtasks were rated as “3, independent” or “N, not applicable”, or when the participant received permission from the medical team to perform the toileting alone even if some subtasks were rated “2, requiring supervision or verbal assistance” or “1, requiring physical assistance” (i.e., if a participant was judged to be independent based on an assessment by the medical team conducted outside the study at irregular intervals based on changes in the patient’s performance; we classified the participant as “independent” even if not all subtasks were rated as “3” at the latest assessment for the study, and the assessment was defined as the endpoint); (*ii*) Mobility change, when participants no longer used a wheelchair because they began to ambulate; (*iii*) Discharge, when participants were discharged from the hospital regardless of the independence level.

Regarding participants’ clinical characteristics on admission, the Stroke Impairment Assessment Set (SIAS) (25, 26), Mini-Mental State Examination-Japanese (MMSE-J) (27, 28), and Functional Independence Measure (FIM) (29, 30) were obtained upon admission to the wards. These assessments have been verified for their reliability and validity in patients post-stroke (25, 26, 31–34).

### Data analysis

Datasets for single participants consisted of independence ratings for each of the 24 subtasks of the TTFA for the number of times the assessment (median, 3 times) was completed. We adopted a two-step cluster analysis to classify the participants into subgroups based on the time course of independence in toileting subtasks. In the cluster analysis, TTAF ratings at 2 time points were used: hospital admission and endpoint. These 2 ratings were combined into a single categorical variable for each subtask (e.g., “1–2” for a participant rated “1” upon admission and “2” at the endpoint). Finally, 24 categorical variables (number of TTAF subtasks) per participant were used in the two-step cluster analysis. In the two-step cluster analysis, several pre-clusters were created based on the distance measure, and smaller clusters were then combined stepwise through a hierarchical cluster analysis. A two-step cluster analysis was employed to automatically determine the number of best-fitting clusters. Log-likelihood was used as the distance measure and Schwarz’s Bayesian criterion was used to determine the cluster number. Subsequently, the clustering quality was evaluated using silhouette coefficients. The coefficients range from -1 to 1, with -1 to 0.2 indicating poor, 0.2 to 0.5 fair, and ≥ 0.5 good (35).

To characterize the clusters, we calculated the percentage of participants corresponding to each of the TTAF ratings (3, 2, 1, and N) for each subtask on admission and at the endpoint for each cluster. To identify participant characteristics for each cluster, demographic and clinical data were compared among the clusters, and for variables that showed significant differences among clusters, multiple comparisons were performed with Bonferroni’s correction. For the nominal scale, Fisher’s exact test was used to compare the clusters, and Fisher’s exact tests were also used for multiple comparisons. For the proportional scale, a one-way ANOVA was performed, and a Student’s t-test was used for multiple comparisons. For the ordinal scale, the Kruskal–Wallis test was performed, and the Mann–Whitney U test was used for multiple comparisons.

Cluster analysis was performed using SPSS version 28 (IBM Corp, Armonk, NY, USA) and subsequent analyses were performed using the R package (R version 4.3.2; R Foundation for Statistical Computing, Vienna, Austria). Differences were considered statistically significant at *p* < 0.05.

## RESULTS

**Fig. 1:**
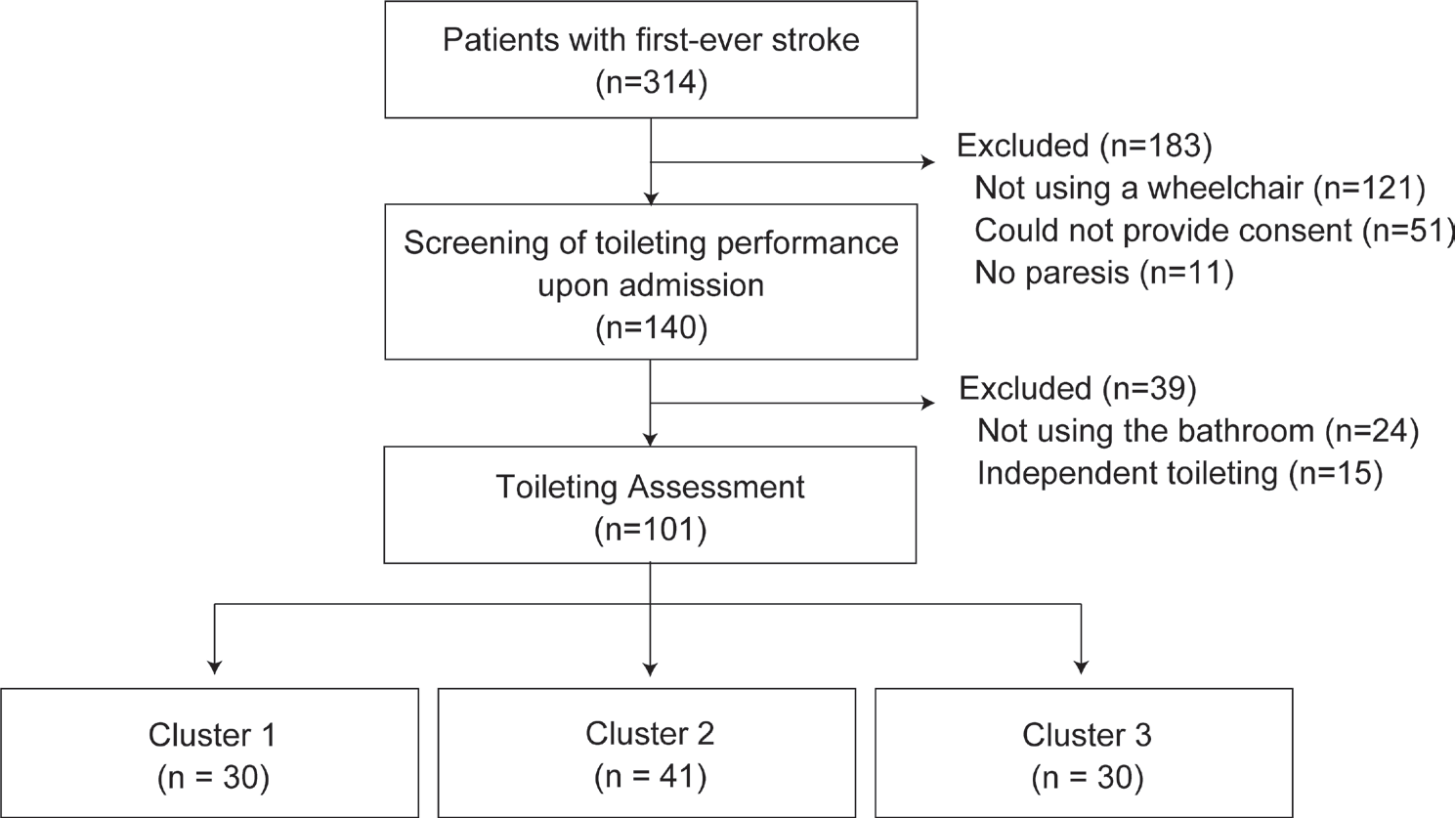
Flowchart of sampling and clustering. The patients admitted with a first stroke during the study period (*n* = 314) were screened upon admission for study eligibility. “Could not provide consent” included the following cases: (*i*) the patient refused to be assessed for toileting; (*ii*) the researcher was unable to contact the legal representative; or (*iii*) the legal representative refused to participate in the study. Notably, researchers approached legal representatives only when the patient was unable to provide informed consent owing to cognitive impairment. Eligible participants (*n* = 101) were classified into 3 clusters by cluster analysis.

Among the 314 patients admitted for their first stroke during the study period, 101 consecutive participants who met the inclusion criteria were included (Fig. 1). The characteristics of the participants are presented in Table II.

**Table II:** Participant characteristics

Item	Cluster 1 (*n* = 30)	Cluster 2 (*n* = 41)	Cluster 3 (*n* = 30)	Comparison of all clusters	Cluster 1 vs Cluster 2	Cluster 1 vs Cluster 3	Cluster 2 vs Cluster 3
				*p*-value			
Age, years, mean (SD)	64.4 (12.2)	70.1 (12.0)	73.4 (14.3)	0.025	0.164	0.033	0.877
Sex, *n* (% in each cluster)							
Male	23 (76.7)	18 (43.9)	14 (46.7)	0.013	0.023	0.098	0.999
Female	7 (23.3)	23 (56.1)	16 (53.3)				
Type of stroke, *n* (% in each cluster)							
Haemorrhage	12 (40.0)	24 (58.5)	8 (26.7)	0.008	0.448	0.134	0.027
Infarction	18 (60.0)	16 (39.0)	17 (56.7)				
Subarachnoid haemorrhage	0 (0.0)	1 (2.4)	5 (16.7)				
Duration after stroke onset at the time of admission, days, mean (SD)	31.1 (14.3)	37.4 (13.7)	44.4 (18.1)	0.004	0.188	0.007	0.200
Paretic sides, *n* (% in each cluster)							
Right	14 (46.7)	26 (63.4)	12 (40.0)	0.127	–	–	–
Left	16 (53.3)	15 (36.6)	18 (60.0)				
Duration of assessment, weeks, mean (SD)	3.1 (2.2)	10.7 (6.1)	6.6 (5.9)	< 0.001	< 0.001	0.011	0.019
Reason for ending the assessment, *n* (% in each cluster)							
Independence of toileting using a wheelchair	20 (66.7)	11 (26.8)	3 (10.0)	< 0.001	< 0.001	< 0.001	0.574
Independence of toileting with changing mobility from wheelchair to walking	9 (30.0)	11 (26.8)	8 (26.7)				
Discharge (not acquiring independence of toileting)	1 (3.3)	19 (46.3)	19 (63.3)				
MMSE-J, median (IQR)	28 (3.5)*	21 (8.5)^†^	18 (10)^‡^	< 0.001	< 0.001	< 0.001	0.999
FIM, median (IQR)							
Motor score	51 (15)	28 (17)	25.5 (20)	< 0.001	< 0.001	< 0.001	0.999
Cognitive score	27 (8.5)	20 (10)	16 (10.5)	< 0.001	0.004	< 0.001	0.103
Total score	76.5 (22)	47 (22)	42.5 (27)	< 0.001	< 0.001	< 0.001	0.761
SIAS, median (IQR)							
Knee–mouth	4 (3)	1 (3)	3 (4)	0.016	0.011	0.999	0.261
Finger function	2.5 (3)	1 (2)	3 (4)	0.028	0.038	0.999	0.151
Hip flexion	3.5 (2)	2 (3)	3 (4)	0.056	–	–	–
Knee extension	4 (2)	2 (3)	3 (4)	0.023	0.013	0.337	0.999
Foot pat	3.5 (2)	2 (3)	2.5 (4)	0.048	0.041	0.707	0.769
Visuospatial	3 (0)	3 (0)^§^	3 (1.5)^||^	0.171	–	–	–
Speech	3 (2)	2 (2)	2 (1)	0.748	–	–	–

The clusters are composed of patients classified based on the results of the TTAF upon their admission and at the assessment endpoint. Cluster 1 showed the highest percentage of “3, independent” ratings for all subtasks at admission and at the end of the assessment. Cluster 2 showed the highest percentage of “1, requiring physical assistance” in all subtasks at admission and the highest percentage of “3, independent” at the endpoint for most subtasks. Cluster 3 showed the highest percentage of “1, requiring physical assistance” in all subtasks upon admission and at the endpoint for most subtasks. MMSE-J, FIM, and SIAS represent results at the time of patient admission. TTAF: Toileting Tasks Assessment Form; IQR: interquartile range; SIAS: Stroke Impairment Assessment Set; MMSE-J: Mini-Mental State Examination-Japanese; FIM: Functional Independence Measure; Missing data:

* = 6,

† =11,

‡ = 8,

§ = 3, and

|| = 6.

**Fig. 2:**
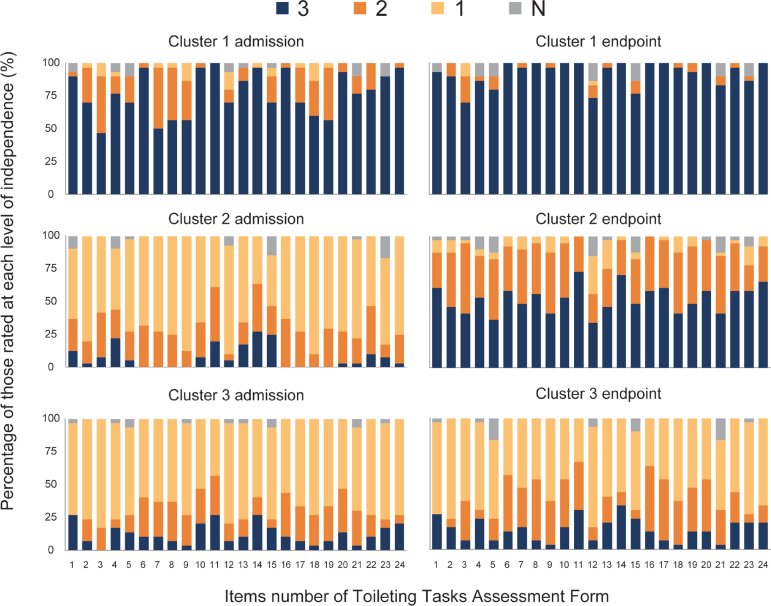
Independence level of each subtask upon admission and at the endpoint in each cluster. The items are listed in order of their performance. Each subtask is assessed as “3, independent”, “2, requiring supervision or verbal assistance”, “1, requiring physical assistance”, and “N, not applicable”.

The participants were classified into 3 clusters by two-step cluster analysis: 30 participants (29.7%) were assigned to Cluster 1, 41 (40.6%) to Cluster 2, and 30 (29.7%) to Cluster 3. Silhouette coefficients were 0.3, indicating that the clustering quality was “fair”. Fig. 2 shows the results of the TTAF assessments for each subtask on admission and at endpoint in each cluster. Cluster 1 showed the highest percentage of “3” ratings for all subtasks at admission (participants rated “3” ranged from 46.7–100% across 24 subtasks; mean percentage of 24 subtasks: 77.2%) and at the endpoint (73.3–100%, mean 92.4%). In 2 subtasks, the percentage of “2” ratings was also as high as that of “3” at the time of admission in the subtasks of “Lock the wheelchair brakes” (46.7% of the participants were rated as “3” and 43.3% as “2”) and “Turn while standing (before urination/defecation)” (50.0% as “3”; 46.7% as “2”). Cluster 2 showed the highest percentage of “1” for all subtasks on admission (participants rated “1” ranged from 39.0–90.2% across 24 subtasks, mean percentage of 24 subtasks: 66.1%) and the highest percentage of “3” for most subtasks (19/24 subtasks) at the endpoint (participants rated “3” from 34.1–73.2% across 24 subtasks, mean percentage of 24 subtasks: 52.6%). In this cluster, the percentage of “2” was also relatively high for all subtasks at the endpoint (participants rated “2” ranged from 22.0–46.3% across 24 subtasks, mean percentage of 24 subtasks: 36.5%), especially for the subtasks of “Lock the wheelchair brakes” (53.7% of the participants were rated as “2”), “Take the foot off the footrest and place it on the ground” (46.3% as “2”), “Pull the lower garments down” (46.3% as “2”), “Pull the lower garments up and adjust them” (46.3% as “2”), and “Put the foot on the footrest” (43.9% as “2”), where the percentage of “2” was higher than that of “3”. Cluster 3 showed the highest percentage of “1” on all subtasks (participants rated “1” ranged from 43.3–83.3% across 24 subtasks, mean percentage of 24 subtasks: 66.8%) upon admission and at the endpoint (participants rated “1” ranged from 33.3–76.7% across 24 subtasks, mean percentage of 24 subtasks: 57.1%) for most subtasks (22/24 subtasks). The subtasks “Stand up from the wheelchair” (43.3% of the participants were rated as “2”), “Maintain a standing position (before urination/defecation)” (46.7% as “2”), “Sit on the toilet seat” (36.7% as “2”), “Stand up from the toilet seat” (50.0% as “2”), and “Maintain a standing position (after urination/defecation)” (46.7% as “2”) had the highest percentage of “2” at the endpoint (3 subtasks had the same percentage as “1”).

**Fig. 3:**
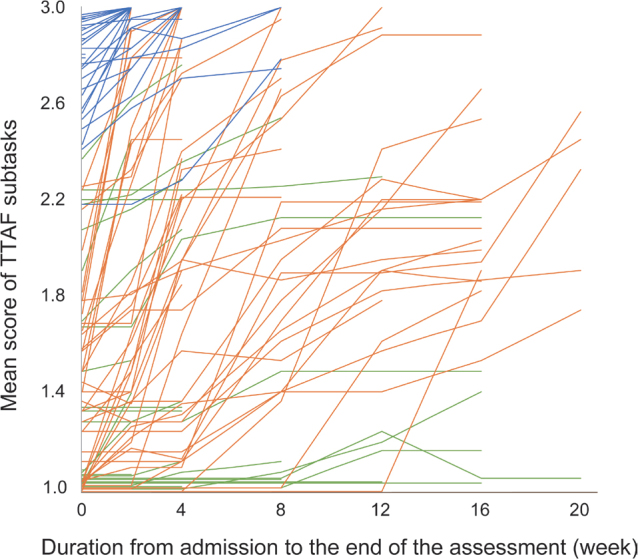
Mean score of TTAF subtasks at each assessment point for individual patients. Each line shows the TTAF scores of individual participants assessed from the time of hospital admission until the criteria for completion of the assessment are met, either every 2 weeks (from the time of admission until the 4th week) or every 4 weeks (after the 4th week). Lines are blue for those in Cluster 1, orange for Cluster 2, and green for Cluster 3. The mean score of TTAF subtasks was calculated by dividing the total score by the number of items excluding those judged “N, not applicable”. TTAF: Toileting Task Assessment Form.

The time course of the mean TTAF subtask scores for individual participants is shown in Fig. 3. Those in Cluster 1 had relatively high scores upon admission and improved their scores over a short period. Those in Cluster 2 had low-to-moderate scores upon admission but improved their scores significantly, while those in Cluster 3 had low-to-moderate scores upon admission and little change in scores during hospitalization.

Table II shows the participant characteristics and the results of the statistical comparisons between the clusters. The following characteristics had statistically significant differences among the 3 clusters: age (*F* [2, 98] = 3.82, *p* = 0.025), sex (*c*^2^ [2] = 8.54, *p* = 0.014), stroke type (*c*^2^ [2] = 30.40, *p* < 0.001), duration after stroke onset (*F* [2, 98] = 5.73, *p* = 0.004), duration of toileting assessment (*F* [2, 98] = 18.75, *p* < 0.001), MMSE-J (*H* [2] = 18.59, *p* < 0.001), FIM (motor: *H* [2] = 33.36, *p* < 0.001, cognitive: *H* [2] = 21.48, *p* < 0.001, and total scores: *H* [2] = 31.28, *p* < 0.001), and SIAS (knee–mouth test: *H* [2] = 8.30, *p* = 0.016, finger-function test: *H* [2] = 7.17, *p* = 0.028, knee-extension test: *H* [2] = 7.56, *p* = 0.023, foot-pat test: *H* [2] = 6.06, *p* = 0.048). In the multiple comparison between Clusters 1 and 2, the participants in Cluster 1 had a higher percentage of males (*p* = 0.023), shorter assessment period (*t* = 6.503, *d* = 4.854, *p* < 0.001), and higher scores on MMSE-J (*U* = 156, *r* = 0.50, *p* < 0.001), FIM (motor: *U* = 160, *r* = 0.63, *p* < 0.001, cognitive: *U* = 337.5, *r* = 0.39, *p* = 0.003, total: *U* = 196, *r* = 0.58, *p* < 0.001), and SIAS (knee–mouth: *U* = 371, *r* = 0.34, *p* = 0.011, finger-function: *U* = 406.5, *r* = 0.30, *p* = 0.038, knee-extension: *U* = 376.5, *r* = 0.34, *p* = 0.013, foot-pat: *U* = 409, *r* = 0.29, *p* = 0.041) than those in Cluster 2. For the comparison between Clusters 1 and 3, those in Cluster 1 were younger (*t* = 2.624, *d* = 13.284, *p* = 0.033), had a shorter duration since stroke onset (*t* = 3.031, *d* = 4.472, *p* = 0.011), shorter assessment period (*t* = 3.031, *d* = 4.471, *p* = 0.011), and higher scores on MMSE-J (*U* = 91, *r* = 0.56, *p* < 0.001) and FIM (motor: *U* = 128.5,*r* = 0.60, *p* < 0.001; cognitive:U = 159.5, *r* = 0.56, *p* < 0.001; total: *U* = 126.5,*r* = 0.62, *p* < 0.001) than those in Cluster 3. In the comparison between Clusters 2 and 3, Cluster 2 had a shorter assessment period (*t* = 2.821, *d* = 6.024, *p* = 0.019) than those in Cluster 3. There was also a significant difference in the percentage of participants with a stroke type (*p* = 0.027), Cluster 2 had more participants with haemorrhage (58.5%), and Cluster 3 had more participants with infarction (56.7%).

The percentage of reasons for ending the assessment (endpoint status) differed among the clusters (*p* < 0.001), with a significant difference between Clusters 1 and 2 (*p* < 0.001) and Clusters 1 and 3 (*p* < 0.001). Cluster 1 had more participants ending the assessment for the reason “independence of toileting” and fewer participants ending the assessment for the reason “discharge” compared with the other clusters.

## DISCUSSION

To understand the time course towards independence in toileting subtasks upon admission, we classified patients with subacute stroke based on the time course of the independence level of subtasks comprising toileting into 3 clusters: Cluster 1, patients who showed near independence in many subtasks upon admission and then became independent early during hospitalization; Cluster 2, patients who required assistance with many subtasks upon admission but could perform many subtasks independently or with supervision/verbal assistance; and Cluster 3, patients requiring assistance upon admission and remaining dependent on discharge. Similar to previous studies showing that the level of independence and changes in overall ADLs can be classified into several different time courses (36–40), we found that the process of acquiring toileting was not uniform and that 3 different time courses existed.

The present study found that patients with stroke could be classified into 3 types based on the time course of their independence level in the toileting subtasks. The characteristics of these 3 time courses were similar to those of the 3 processes towards independence of the subtasks comprising bed–wheelchair transfer (22). Considering that bed–wheelchair transfer and toileting using a wheelchair – which have different difficulty levels for independence (6–9) – had similar processes towards independence, the existence of multiple subtypes of the process towards independence may be a common characteristic of the serial ADL skills. When considering intervention strategies for such tasks, it is necessary to set specific goals for each subtype, e.g., which subtask to target and to what extent improvement is required, as the contribution on the independence level of each subtask differs depending on each subtype.

Comparing patient characteristics across clusters, participants in Cluster 1 – which had the highest percentage of participants who were independent in the subtasks – were younger, had a higher percentage of males, a shorter time since stroke onset, higher motor and cognitive function, and greater functional independence than those in Clusters 2 and 3. Previous studies have reported that factors predicting improvement in ADL include male sex (41–44), younger age (41, 45–47), mild motor (42, 48) and cognitive impairment (43, 48), and shorter post-onset period (41, 49). These factors were consistent with the characteristics of Cluster 1 in the present study. Patients with these characteristics retained the ability to learn ADL tasks and could be expected to become independent in many toileting subtasks during early hospitalization.

Cluster 2 had more participants with haemorrhage and Cluster 3 more participants with infarction; the difference in the percentage of independent participants in the subtasks at the endpoint in the 2 clusters could be due to the different recovery potentials of these 2 stroke types. Functional recovery during the acute to subacute phase is greater in patients with cerebral haemorrhage than in those with cerebral infarction (50–52). Therefore, it is plausible that Cluster 2 had more participants who were independent in each subtask at the endpoint than Cluster 3. This may be because Cluster 2 included more participants with a higher potential for recovery (i.e., participants after cerebral haemorrhage). Additionally, Cluster 2 was assessed for a longer time; therefore, these patients had more time to practise to achieve toileting independence. This may be because these patients demonstrated significant recovery during their hospitalization, leading to expectations of continued improvement; consequently, their hospital stay (consequently, their rehabilitation) was extended. This may have facilitated further recovery. In fact, patients in Cluster 2 demonstrated greater improvement in overall toileting performance since admission compared with those in Cluster 3.

Participants in Cluster 1 who were independent in many subtasks upon admission were more likely to become independent in toileting when using a wheelchair early during hospitalization. Until patients achieve independence, they often require assistance or supervision because toileting is an activity with a high risk of falling (53–56). In contrast, achieving toileting independence allows patients to urinate or defecate in a bathroom in their own time without having to call staff, which leads to increased activity in the ward. Therefore, it is important to encourage patients to become independent as early as possible to increase their activity levels and rebuild their lives. To develop strategies to facilitate early patient independence, identifying non-independent subtasks early in hospitalization and assessing their independence levels would be helpful. It is particularly important to evaluate performance early after hospital admission and facilitate early learning of movements for subtasks such as “Lock the wheelchair brakes” and “Turn while standing (before urination/defecation)”, for which the percentage of “2, requiring supervision or verbal assistance” was high.

Participants in Cluster 2, who required assistance with many subtasks early in their hospitalization, but whose overall toileting independence improved over the course of their hospitalization, had a high probability of being able to perform many subtasks independently or with supervision and verbal assistance during prolonged hospitalization. Therefore, intervention strategies for patients in this group should be designed to achieve independence in all subtasks. In particular, it is important to facilitate the acquisition of subtasks related to manipulating lower garments and a wheelchair, which had a high percentage of “2” ratings at the endpoint. If repeated practice does not lead to success in these subtasks, considering compensatory methods such as environmental adjustment and assistive devices (57, 58) is recommended rather than endlessly practising until independence is achieved. Alternatively, considering that half of the participants with independent toileting walked without a wheelchair, patients with high ambulatory ability but difficulty in wheelchair manipulation may achieve early mobility independence by switching from wheelchair to walking; this eliminates the need to master wheelchair manipulation. Conversely, the subtasks related to the manipulation of lower garments are essential to perform toileting, regardless of mobility means or wheelchair use. In many cases, the patient must manipulate the lower garments in a standing position, which requires high balance function (59). Therefore, it may be advisable to put effort into balance training in the standing position early to ensure sufficient practice.

Participants in Cluster 3 who required assistance with many subtasks upon admission and whose independence level changed slightly during subsequent hospitalization had a high probability of requiring assistance with many subtasks during hospitalization. Because patients in this cluster are expected to have difficulty in becoming independent, the focus of practice should be on subtasks that may reduce the required assistance. Assisting with toileting is highly stressful for both patients and their caregivers (1, 2). Therefore, a practice strategy should be developed based on identifying the specific needs of the patient and their caregiver and evaluating which subtasks would be less burdensome if the patient’s independence level is improved. In addition, regarding task difficulty, the subtasks of standing up and maintaining a sitting or standing position had a high percentage of “2” ratings at the endpoint and were relatively easy to improve towards better independence. Intensive practice of these subtasks is expected to provide a relatively large return on the effort put into practice.

This study has some limitations. First, the present study did not have a large sample size. A larger sample may be needed to assess the variables for the cluster analysis of the present study (60). Therefore, for more robust results and validation, a larger sample size should be used. Second, the present study does not reflect the process of acquiring toileting in patients with a severe impairment that prevents them from transferring to a toilet seat upon admission because patients who did not urinate or defecate in the bathroom were not included. Therefore, when interpreting these results, it should be noted that they do not show a trend for all patients after stroke who require rehabilitation to acquire toileting. Third, the degree of influence of each patient’s characteristics on clustering is unclear, and the time course of acquiring toileting skills during hospitalization (i.e., determining the cluster to which the patient belongs) cannot be predicted from their status on admission, which would enable more tailored interventions for individual patients. This important aspect may be addressed in future studies focusing on developing a model to identify the clusters to which patients belong. Fourth, the TTAF broadly assesses a patient’s need for assistance but cannot identify why they need assistance (i.e., whether it is because of a motor or cognitive impairment). Using specific tools (61, 62) to assess and clarify the factors that influence a patient’s level of independence, such as cognitive deficits, would indicate the type of intervention that would be appropriate for each patient characteristic. Finally, as this was a single-centre study, the results may have been influenced by facility-specific conditions, such as the criteria for determining patient independence and the hospital environment. In addition, because the target population was limited to patients admitted to a rehabilitation hospital, changes in toilet performance during the acute phase and after discharge remain unclear. Consequently, the present results may not fully represent the overall time course of toilet performance. Future studies should investigate changes in toileting performance during the acute and chronic phases of individuals after stroke – including in those with severe dysfunction – in a multicentre setting to identify the specific changes occurring in each phase. Understanding the overall process of acquiring toileting ability after stroke would provide valuable insights for developing more specific intervention strategies in each phase.

In conclusion, the level of independence in toileting and its changing processes was classified into 3 different time courses, each of which may have different effective intervention strategies. The findings of this study can be used to prioritize subtasks to be practised depending on each patient’s time course of toileting independence in the early stages of hospitalization.

## Supplementary Material

TIME COURSE FOR ACQUIRING TOILETING INDEPENDENCE IN PATIENTS WITH SUBACUTE STROKE: A PROSPECTIVE COHORT STUDY
