# Supplementary material for: TIME COURSE FOR ACQUIRING TOILETING INDEPENDENCE IN PATIENTS WITH SUBACUTE STROKE: A PROSPECTIVE COHORT STUDY
Source: J Rehabil Med. 2025 May 20;57:42390. doi: 10.2340/jrm.v57.42390 (PMC12108136; doi:10.2340/jrm.v57.42390)
Supplement: TIME COURSE FOR ACQUIRING TOILETING INDEPENDENCE IN PATIENTS WITH SUBACUTE STROKE: A PROSPECTIVE COHORT STUDY [file JRM-57-42390-s1.pdf]

Toileting Tasks Assessment Form (TTAF)

General comments:

Patient name: Assessor: Date: ～ Time of the day:

Toilet: ☐Toilet in the ward ☐Portable toilet Score: 3, independent; 2, requires supervision or verbal assistance; 1, requires assistance; N, not applicable

		Task	Score	Comments
Wheelchair to the toilet seat	Approach to the toilet	1. Open and close the door		
		2. Maneuver the wheelchair towards the appropriate place for transfer to the toilet seat		
	Transfer	3. Lock the wheelchair brakes		
		4. Press the nurse call button		
		5. Take the foot off the footrest and place it on the ground		
		6. Stand up from the wheelchair		
		7. Turn while standing		
	Pull the lower garments down	8. Maintain a standing position		
		9. Pull the lower garments down		
Performance on toilet seat	Transfer	10. Sit on the toilet seat		
		11. Maintain a sitting position on the toilet seat		
	Clean up	12. Dispose incontinence pad/sanitary items		
		13. Clean up after urination and/or defecation		
		14. Flush the toilet		
		15. Press the nurse call button		
Toilet seat to the wheelchair	Transfer	16. Stand up from the toilet seat		
	Pull the lower garments up	17. Maintain a standing position		
		18. Pull the lower garments up and adjust them		
	Transfer	19. Turn while standing		
		20. Sit on the wheelchair seat		
		21. Put the foot on the footrest		
		22. Unlock the wheelchair brakes		
	Get out of the toilet	23. Open and close the door		
		24. Exit the toilet room		
